# Dose-dependent association between physical activity and mental health, and mitigation effects on risk behaviors

**DOI:** 10.1016/j.isci.2025.111866

**Published:** 2025-01-23

**Authors:** Huixuan Zhou, Feng Jiang, Huanzhong Liu, Yibo Wu, Yi-lang Tang

**Affiliations:** 1 Department of Physical Fitness and Health, School of Sport Science, Beijing Sport University, Beijing 100084, China; 2 Key Laboratory of Exercise and Physical Fitness, Ministry of Education, Beijing Sport University, Beijing 100084, China; 3 School of International and Public Affairs, Shanghai Jiao Tong University, Shanghai 200030, China; 4 Institute of Healthy Yangtze River Delta, Shanghai Jiao Tong University, Shanghai 200030, China; 5 Department of Psychiatry, Chaohu Hospital of Anhui Medical University, Hefei 238000, China; 6 Department of Psychiatry, School of Mental Health and Psychological Sciences, Anhui Medical University, Hefei 230032, China; 7 Anhui Psychiatric Center, Hefei 230022, China; 8 School of Public Health, Peking University, Beijing 100191, China; 9 Department of Psychiatry and Behavioral Sciences, Emory University, Atlanta, GA 30329, USA; 10 Substance Abuse Treatment Program, Atlanta Veterans Affairs Medical Center, Decatur, GA 30033, USA

**Keywords:** Public health, Kinesiology, Psychology

## Abstract

Understanding the dose-response effects of physical activity on mental health and risk behavior mitigation is crucial for mental health promotion. This study using restricted cubic spline and piecewise regression analyses based on a representative national sample of 30,054 Chinese adults, revealed reverse J-shaped (*p* for nonlinear <0.001) but monotonic beneficial associations between physical activity and depression, anxiety, and stress symptoms, with optimal thresholds identified at 2.15 METs-hour/day for depression and anxiety, and 3.25 METs-hour/day for stress. Engaging in 1–3 METs-hour/day of physical activity appeared to mitigate the adverse effects of unhealthy food intake on depression and anxiety, whereas 4–6 METs-hour/day could offset the impact of short sleep duration on depression, anxiety, and stress. The findings suggest that physical activity prescription could be effective in mitigating the adverse effects of certain risk behaviors on common mental symptoms, and excessive physical activity might not be necessary for mental health promotion.

## Highlights

• Physical activity has a nonlinear but beneficial relationship with mental health
• Excessive physical activity is not necessary for mental health
• Beneficial effects of physical activity vary with the type of mental symptoms
• Adverse impact of risk behaviors can be partially mitigated by physical activity

## Graphical abstract

**Figure:**
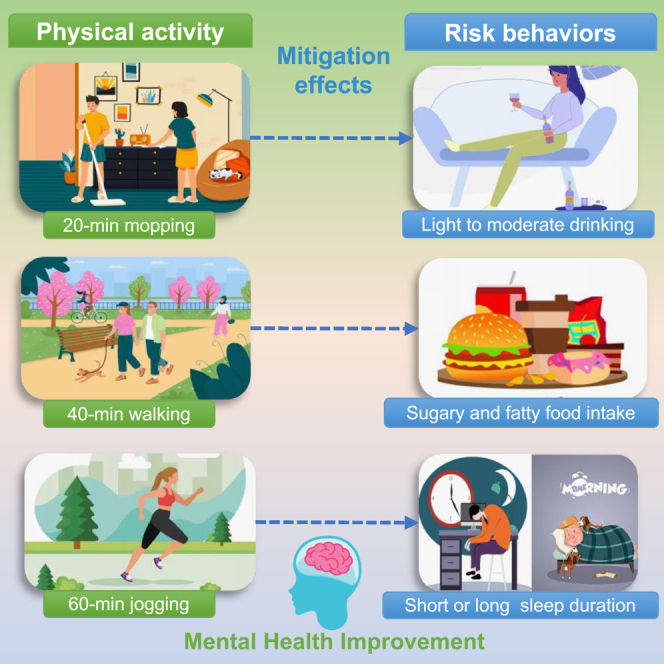

## Introduction

Mental disorders, such as depression, anxiety, and substance use disorders, are major contributors to the global burden of disease.^1^^,^^2^ They impair the mental health of millions of people worldwide, and nearly one-sixth live in China.^2^ The COVID-19 pandemic has also been linked to increased rates of mental disorders.^3^^,^^4^ Health behavior interventions are low-cost strategies to improve mental health.^5^^,^^6^ Consequently, exploring the associations between health behaviors and mental health can inform future interventions.

It has been established that physical activity is beneficial for symptoms of depression and anxiety.^7^ However, some cross-sectional studies on the dose-response relationship between physical activity and mental health, which describe the magnitude of changes in mental health outcomes as a function of physical activity quantity,^8^ have reported nonlinear associations and inconsistent findings. Studies on Canadian adults^9^ and adolescents from Europe and North America^10^ find beneficial and curvilinear relationships between physical activity and general mental health. In contrast, studies on US adults^11^^,^^12^ show an inverted U-shaped relationship, suggesting that excessively long exercises may worsen mental health. These findings raise the question of whether more physical activity is always better.

One possible source of inconsistency is the use of various outcome measurements. Instead of using standardized rating scales for specific symptoms of mental disorders, those studies relied on a single-item question about general mental health, which may introduce some randomness in data collection and obscure the associations between physical activity and common mental disorders.^13^ Another potential confounder is the omission of health behaviors that may be associated with mental health.^10^^,^^11^ Systematic reviews and some longitudinal studies have shown that poor eating habits and tobacco use are associated with a higher risk of mental symptoms,^14^^,^^15^ while alcohol use has an interactive relationship with mental health,^16^^,^^17^ and improved sleep is causally related to the improvement of mental symptoms such as depression, anxiety, and stress.^18^ Tian et al. clarified that eating habits, smoking, alcohol drinking, and sleep quality affect mental health mainly by influencing the physiology of musculoskeletal and cardiovascular systems, as well as the volume of brain gray matter.^19^ Therefore, using standardized rating scales for mental symptoms and adjusting health behaviors as confounders may help obtain more reliable conclusions about the relationship between physical activity and mental health.

Moreover, risk behaviors generally cluster with physical activity,^20^^,^^21^^,^^22^ partially because people tend to increase the amount of physical activity to mitigate the potential adverse effects of unhealthy habits such as smoking and drinking.^23^^,^^24^ Zhang et al.’s study on the interactive effects of physical activity and sleep shows that physical activity could decrease negative emotions in poor sleepers, but has no significant effects in people with healthy sleep patterns.^25^ However, the dose of physical activity needed to compensate for the adverse effects of risk behaviors on different mental symptoms remains unexplored.

Given that previous studies on dose-response relationships between physical activity and mental health show inconsistent results, and few studies have examined the dose of physical activity that could compensate for the adverse effects of risk behaviors on various mental symptoms using advanced regression techniques, this study using restricted cubic spline (RCS) regression and piecewise regression analyses to: 1) examine the dose-response relationships between physical activity and several specific mental health symptoms, 2) assess the dose of physical activity that could mitigate the adverse effects of other risk behaviors (unhealthy food intake, smoking, drinking, short and long sleep duration) on mental health. Utilizing a nationally representative dataset from China, a country facing a substantial burden of mental disorders, this study uses several mental health symptoms assessed by standardized rating scales and health behaviors potentially associated with mental health. The study findings will contribute to understanding the relationships between physical activity and mental health outcomes, which is one of the vital areas of physical activity epidemiology. Moreover, this study could promote public awareness about the importance of daily health behaviors in mental well-being, and provide evidence-based guidance for policymakers and mental health organizations in China and beyond to tailor programs and interventions toward mental health promotion.

## Results

Of 30,054 adults in Psychology and Behavior Investigation of Chinese Residents in 2023, with a mean (SD) age of 43.0 (16.6) years, 15,043 (50.1%) were female (Table 1), 4938 (16.4%) were smokers, and 7457 (24.8%) were alcohol drinkers; 15,435 (51.4%) endorsed depression, 12,575 (41.8%) endorsed anxiety, and 15,851 (52.7%) endorsed stress symptoms, respectively, and they were less physically active than those who did not have these symptoms (Table 2). Among smokers, 1642 (33.3%) had high nicotine dependence, and among alcohol drinkers, 1259 (16.9%) reported the experiencing alcohol withdrawal anxiety when they stopped drinking. Tables S1 and S2 in the supplemental information show how the characteristics differed by mental health outcomes.

**Table 1:** Basic characteristics of the whole study sample (*n* = 30054)

Characteristics	Total sample (%)
Age (year)^a^	43.0 **±** 16.6
Sex	
Male	15011 (49.9)
Female	15043 (50.1)
Education level	
Primary or middle school	9243 (30.8)
High school	6187 (20.6)
Technical school	4010 (13.3)
University	10614 (35.3)
Employment status	
Employed	13302 (44.3)
Students	4681 (15.6)
Retired	4228 (14.1)
Self-employed	5043 (16.8)
Unemployed	2800 (9.3)
Monthly family income per capita^b^	
Very low (<2000 CNY)	8781 (29.2)
Low (2001–3000 CNY)	9460 (31.5)
Middle (3001–4000 CNY)	3968 (13.2)
High (4001–6000 CNY)	3491 (11.6)
Very high (>6000 CNY)	4354 (14.5)
Marital status	
Married	19427 (64.6)
Others	10627 (35.4)
Place of residence	
Rural	9319 (31.0)
Urban	20735 (69.0)
Presence of any chronic medical disorders	
Yes	9594 (31.9)
No	20460 (68.1)
BMI (kg/m^2^)^a^	22.4 **±** 3.4

BMI, body mass index.

a Data are presented as mean ± SD.

b According to the China Statistical Yearbook, the average per capita monthly income of Chinese residents in 2022 was 3073 CNY (428 USD).

**Table 2:** Physical activity by various risk behaviors and mental health outcomes (*n* = 30054)

	Total sample (%)	Physical activity, METs-hour per day, mean ± SD	H/t statistic	*p* value
Total	30054 (100)	4.9 **±** 5.8		
Unhealthy food intake (>3 times per week)			53.96	<0.001
No	17287 (57.5)	4.7 **±** 5.7		
Sugary food	5235 (17.4)	5.0 **±** 5.8		
Fatty food	3418 (11.4)	4.9 **±** 5.3		
Both of sugary and fatty food	4114 (13.7)	5.1 **±** 6.1		
Smoking			43.21	<0.001
No	25116 (83.6)	4.7 **±** 5.6		
Light (≤10 cigarettes per day)	2926 (9.7)	5.3 **±** 6.5		
Moderate (11–20 cigarettes per day)	1450 (4.8)	5.7 **±** 6.5		
Heavy (>20 cigarettes per day)	562 (1.9)	5.5 **±** 7.0		
Drinking			228.06	<0.001
No	22597 (75.2)	4.6 **±** 5.4		
Light (≤50mL per day)	3439 (11.4)	5.3 **±** 6.2		
Moderate (51–150mL per day)	1703 (5.7)	5.5 **±** 6.1		
Heavy (>150mL per day)	2315 (7.7)	6.4 **±** 7.4		
Short sleep duration			116.40	<0.001
No (7–8 h per night and longer)	22354 (74.4)	4.9 **±** 5.7		
Light (6 h per night)	5228 (17.4)	5.0 **±** 5.9		
Moderate (5 h per night)	1288 (4.3)	5.0 **±** 6.0		
Extreme (≤4 h per night)	1184 (3.9)	4.2 **±** 6.3		
Long sleep duration			37.09	<0.001
No (7–8 h per night and shorter)	25861 (86.0)	4.9 **±** 5.7		
Light (9 h per night)	2745 (9.1)	4.5 **±** 5.5		
Moderate (10 h per night)	1037 (3.5)	5.0 **±** 6.9		
Extreme (≥11 h per night)	411 (1.4)	6.0 **±** 8.9		
Depression			3.80	<0.001
Yes	15435 (51.4)	4.7 ± 5.7		
No	14619 (48.6)	5.0 ± 5.8		
Anxiety			4.43	<0.001
Yes	12575 (41.8)	4.7 ± 5.7		
No	17479 (58.2)	5.0 ± 5.8		
Stress			5.08	<0.001
Yes	15851 (52.7)	4.7 ± 5.7		
No	14203 (47.3)	5.0 ± 5.8		
Nicotine dependence	4938 (1000)		−0.36	0.721
High	1642 (33.3)	5.5 ± 7.0		
Low	3296 (66.7)	5.5 ± 6.4		
Alcohol withdrawal anxiety	7457 (100)		0.97	0.328
Yes	1259 (16.9)	5.5 ± 6.7		
No	6198 (83.1)	5.7 ± 6.6		

MET, metabolic equivalent. H is the statistic for the Kruskal-Wallis test. T is the statistic for the Student’s t test.

The mean (SD) physical activity score was 4.9 (5.8) METs-hour per day, and 17,299 (57.6%) had a moderate level of physical activity, while 7916 (26.3%) had a high level, and 4839 (16.1%) had a low level. Participants who usually ate unhealthy foods, who were smokers or drinkers, and people with short or extremely long sleep duration tended to have higher physical activity scores than those who did not; people who slept very short hours were less physically active than those who slept longer (Table 2).

### Dose-dependent association between physical activity and various mental health symptoms

**Figure 1:**
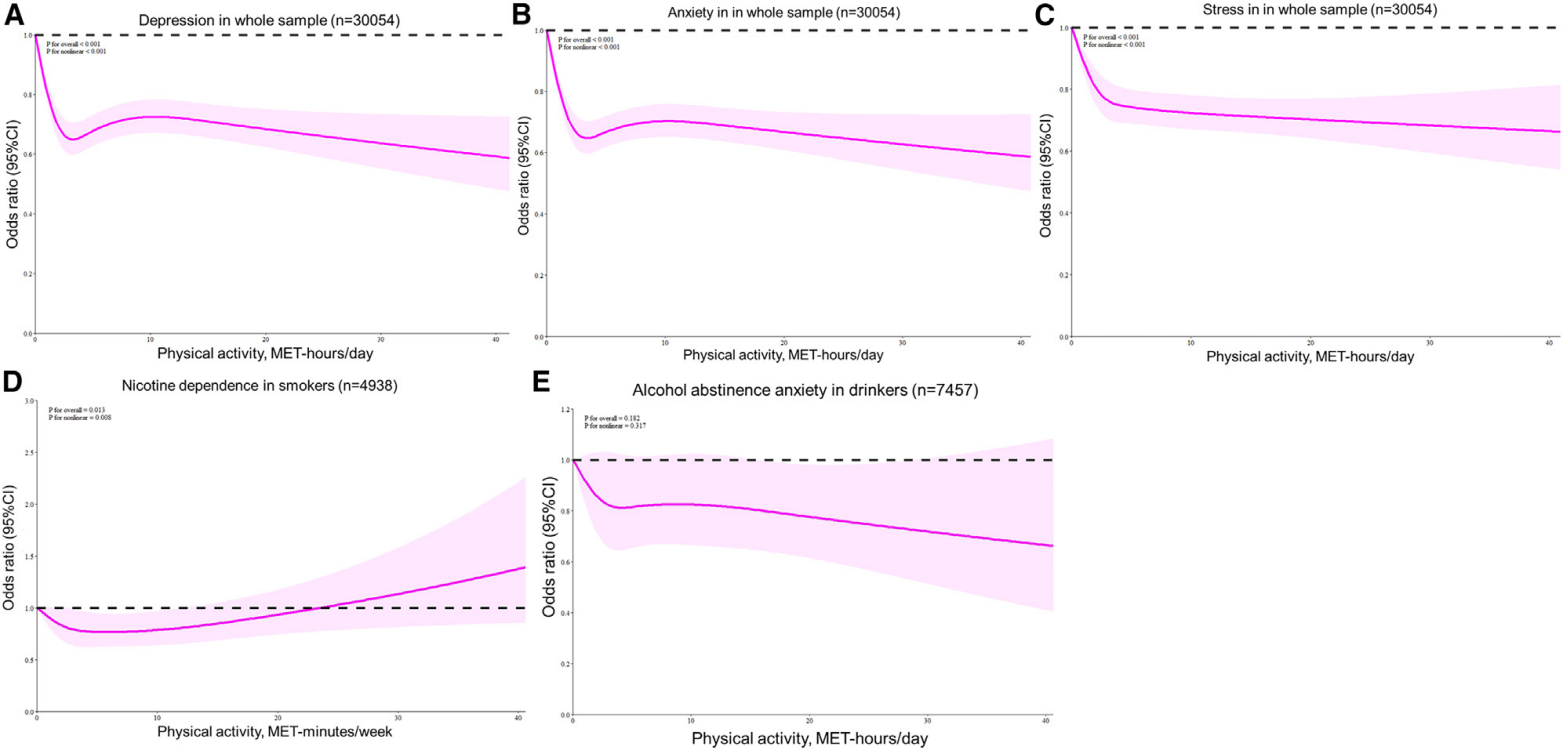
Dose-response associations between physical activity and mental health outcomes from restricted cubic spline regression (A) Depression, *p* for nonlinear <0.001; (B) anxiety, *p* for nonlinear <0.001; (C) stress, *p* for nonlinear <0.001; (D) nicotine dependence, *p* for nonlinear = 0.008; (D) alcohol withdrawal anxiety, *p* for nonlinear = 0.317. The estimates were adjusted for covariates and risk behaviors.

(1) Depression: As shown in Figure 1A, the results from the RCS model revealed that physical activity had a reverse J-shaped association with depression (*p* for nonlinear <0.001). According to the piecewise model (Table 3), physical activity was significantly associated with a lower likelihood of depression (OR = 0.81, 95%CI: 0.77–0.85, *p* < 0.001), up to a threshold of 2.15 METs-hour per day. However, physical activity did not have a significant effect on depression when it surpassed 2.15 METs-hour per day (OR = 1.00, 95%CI: 1.00–1.01, *p* = 0.907) (Table 3).

**Table 3:** Association between physical activity and depression below and above the threshold

	PA≤2.15 (*n* = 10855)					PA>2.15 (*n* = 19199)				
	**β**	*p****value***	**OR**	**Lower 95%CI**	**Upper 95%CI**	**β**	*p****value***	**OR**	**Lower 95%CI**	**Upper 95%CI**
PA, METs-hour per day	−0.21	<0.001	0.81	0.77	0.85	0.00	0.907	1.00	1.00	1.01
Unhealthy food intake, ref. no										
Sugary food	0.40	<0.001	1.49	1.33	1.68	0.43	<0.001	1.53	1.41	1.67
Fatty food	0.62	<0.001	1.86	1.62	2.14	0.57	<0.001	1.77	1.61	1.95
Both of sugary and fatty food	0.66	<0.001	1.93	1.69	2.21	0.53	<0.001	1.69	1.54	1.86
Smoking, ref. no										
Light (≤10 cigarettes per day)	−0.05	0.487	0.95	0.82	1.10	0.05	0.410	1.05	0.94	1.16
Moderate (11–20 cigarettes per day)	0.09	0.414	1.09	0.88	1.35	0.16	0.034	1.17	1.01	1.35
Heavy (>20 cigarettes per day)	0.32	0.054	1.38	0.99	1.91	0.18	0.125	1.20	0.95	1.52
Drinking, ref. no										
Light (≤50mL per day)	0.17	0.014	1.18	1.03	1.35	0.20	<0.001	1.22	1.11	1.34
Moderate (51–150mL per day)	0.12	0.247	1.12	0.92	1.37	0.09	0.182	1.09	0.96	1.24
Heavy (>150mL per day)	0.18	0.057	1.20	0.99	1.44	0.04	0.493	1.04	0.93	1.17
Short sleep duration, ref. no										
Light (6 h per night)	0.57	<0.001	1.77	1.58	1.97	0.42	<0.001	1.52	1.40	1.65
Moderate (5 h per night)	0.79	<0.001	2.19	1.78	2.70	0.73	<0.001	2.08	1.79	2.42
Extreme (≤4 h per night)	1.05	<0.001	2.87	2.32	3.55	1.25	<0.001	3.50	2.86	4.29
Long sleep duration, ref. no										
Light (9 h per night)	−0.07	0.314	0.93	0.82	1.07	−0.08	0.159	0.93	0.83	1.03
Moderate (10 h per night)	0.41	<0.001	1.51	1.23	1.85	0.33	<0.001	1.39	1.17	1.64
Extreme (≥11 h per night)	0.72	<0.001	2.06	1.45	2.91	0.83	<0.001	2.29	1.72	3.04

The estimates were adjusted for covariates. PA, physical activity; MET, metabolic equivalent; ref., reference group; OR, odds ratio; CI, confidence interval.
(2) Anxiety: Physical activity had a reverse J-shaped association with anxiety (*p* for nonlinear <0.001) (Figure 1B), and was significantly associated with a lower likelihood of anxiety (OR = 0.77, 95%CI: 0.73–0.81, *p* < 0.001) up to a threshold of 2.15 METs-hour per day (Table 4). The association became insignificant over the threshold (OR = 1.00, 95%CI: 0.99–1.00, *p* = 0.432) (Table 4).

**Table 4:** Association between physical activity and anxiety below and above the threshold

	PA≤2.15 (*n* = 10855)					PA>2.15 (*n* = 19199)				
	**β**	*p****value***	**OR**	**Lower 95%CI**	**Upper 95%CI**	**β**	*p****value***	**OR**	**Lower 95%CI**	**Upper 95%CI**
PA, METs-hour per day	−0.26	<0.001	0.77	0.73	0.81	−0.002	0.432	1.00	0.99	1.00
Unhealthy food intake, ref. no										
Sugary food	0.32	<0.001	1.38	1.23	1.55	0.38	<0.001	1.47	1.35	1.60
Fatty food	0.55	<0.001	1.73	1.51	1.98	0.55	<0.001	1.74	1.58	1.91
Both of sugary and fatty food	0.62	<0.001	1.86	1.64	2.12	0.59	<0.001	1.81	1.65	1.98
Smoking, ref. no										
Light (≤10 cigarettes per day)	−0.12	0.117	0.89	0.76	1.03	0.09	0.114	1.09	0.98	1.22
Moderate (11–20 cigarettes per day)	0.11	0.304	1.12	0.91	1.38	0.16	0.027	1.18	1.02	1.36
Heavy (>20 cigarettes per day)	0.02	0.890	1.02	0.75	1.39	0.10	0.407	1.10	0.88	1.39
Drinking, ref. no										
Light (≤50mL per day)	0.15	0.022	1.17	1.02	1.33	0.12	0.013	1.13	1.03	1.24
Moderate (51–150mL per day)	0.08	0.412	1.09	0.89	1.32	0.04	0.508	1.04	0.92	1.19
Heavy (>150mL per day)	0.10	0.302	1.10	0.92	1.32	0.12	0.041	1.13	1.00	1.26
Short sleep duration, ref. no										
Light (6 h per night)	0.49	<0.001	1.63	1.46	1.81	0.37	<0.001	1.45	1.34	1.57
Moderate (5 h per night)	0.72	<0.001	2.05	1.68	2.49	0.65	<0.001	1.91	1.65	2.21
Extreme (≤4 h per night)	1.09	<0.001	2.98	2.44	3.62	1.12	<0.001	3.08	2.56	3.70
Long sleep duration, ref. no										
Light (9 h per night)	−0.06	0.399	0.94	0.82	1.08	−0.08	0.135	0.92	0.82	1.03
Moderate (10 h per night)	0.30	0.004	1.35	1.10	1.64	0.31	<0.001	1.36	1.15	1.62
Extreme (≥11 h per night)	0.54	0.001	1.72	1.25	2.37	0.85	<0.001	2.34	1.78	3.06

The estimates were adjusted for covariates. PA, physical activity; MET, metabolic equivalent; ref., reference group; OR, odds ratio; CI, confidence interval.
(3) Stress: Physical activity had a reverse J-shaped association with stress (*p* for nonlinear <0.001) (Figure 1C), and was significantly associated with a lower likelihood of stress (OR = 0.89, 95%CI: 0.86–0.92, *p* < 0.001), up to a threshold of 3.25 METs-hour per day (Table 5). The association became insignificant over the threshold (OR = 1.00, 95%CI: 0.99–1.00, *p* = 0.125) (Table 5).

**Table 5:** Association between physical activity and stress below and above the threshold

	PA<3.25 (n = 15033)					PA>3.25 (n = 15021)				
	**β**	*p****value***	**OR**	**Lower 95%CI**	**Upper 95%CI**	**β**	*p****value***	**OR**	**Lower 95%CI**	**Upper 95%CI**
PA, METs-hour per day	−0.12	<0.001	0.89	0.86	0.92	−0.004	0.125	1.00	0.99	1.00
Unhealthy food intake, ref. no										
Sugary food	0.33	<0.001	1.39	1.26	1.53	0.40	<0.001	1.49	1.36	1.64
Fatty food	0.52	<0.001	1.68	1.50	1.89	0.50	<0.001	1.65	1.49	1.84
Both of sugary and fatty food	0.55	<0.001	1.73	1.55	1.93	0.55	<0.001	1.73	1.56	1.91
Smoking, ref. no										
Light (≤10 cigarettes per day)	−0.16	0.014	0.85	0.75	0.97	0.00	0.963	1.00	0.89	1.12
Moderate (11–20 cigarettes per day)	−0.23	0.012	0.80	0.67	0.95	0.01	0.919	1.01	0.86	1.18
Heavy (>20 cigarettes per day)	−0.12	0.368	0.89	0.68	1.15	−0.02	0.859	0.98	0.76	1.26
Drinking, ref. no										
Light (≤50mL per day)	0.25	<0.001	1.29	1.15	1.44	0.13	0.021	1.13	1.02	1.26
Moderate (51–150mL per day)	0.17	0.039	1.18	1.01	1.38	0.13	0.074	1.14	0.99	1.32
Heavy (>150mL per day)	0.31	<0.001	1.37	1.18	1.59	0.20	0.001	1.22	1.08	1.38
Short sleep duration, ref. no										
Light (6 h per night)	0.48	<0.001	1.62	1.48	1.78	0.15	0.001	1.17	1.07	1.27
Moderate (5 h per night)	0.69	<0.001	1.99	1.67	2.37	0.20	0.018	1.22	1.03	1.44
Extreme (≤4 h per night)	0.64	<0.001	1.90	1.59	2.28	0.79	<0.001	2.20	1.79	2.71
Long sleep duration, ref. no										
Light (9 h per night)	0.01	0.808	1.01	0.91	1.14	−0.16	0.010	0.85	0.76	0.96
Moderate (10 h per night)	0.30	0.001	1.36	1.14	1.62	0.11	0.266	1.11	0.92	1.35
Extreme (≥11 h per night)	0.63	<0.001	1.87	1.39	2.52	0.49	0.002	1.63	1.20	2.21

The estimates were adjusted for covariates. PA, physical activity; MET, metabolic equivalent; ref., reference group; OR, odds ratio; CI, confidence interval.
(4) Nicotine dependence: Physical activity had a U-shaped association with nicotine dependence (*p* for nonlinear = 0.008) (Figure 1D). Physical activity showed a marginally significant inverse association with nicotine dependence in smokers (OR = 0.94, 95%CI: 0.88–1.00, p = 0.047), with a threshold of 4.26 METs-hour per day, and showed a marginally significant direct association with nicotine dependence over the threshold (OR = 1.02, 95%CI: 1.01–1.03, *p* = 0.005) (Table S3).
(5) Alcohol withdrawal anxiety: There was no nonlinear association between physical activity and alcohol withdrawal anxiety (*p* for nonlinear = 0.317) (Figure 1E), and physical activity did not have a significant effect on alcohol withdrawal anxiety in drinkers (OR = 0.99, 95%CI: 0.98–1.00, *p* = 0.114) (Table S4).

### Level of physical activity for risk behavior mitigation

As shown in Tables 3, 4, 5, S3, and S4, unhealthy food intake, and short and long sleep durations were significantly associated with a higher likelihood of all five mental health symptoms. Drinking was significantly associated with a higher likelihood of common mental health symptoms such as depression, anxiety, and stress, as well as alcohol withdrawal anxiety. Smoking was significantly associated with a higher likelihood of nicotine dependence and alcohol withdrawal anxiety, but not with depression and anxiety. A significant association was also found between smoking and a lower likelihood of stress, up to the physical activity threshold of 3.25 METs-hour per day.

**Figure 2:**
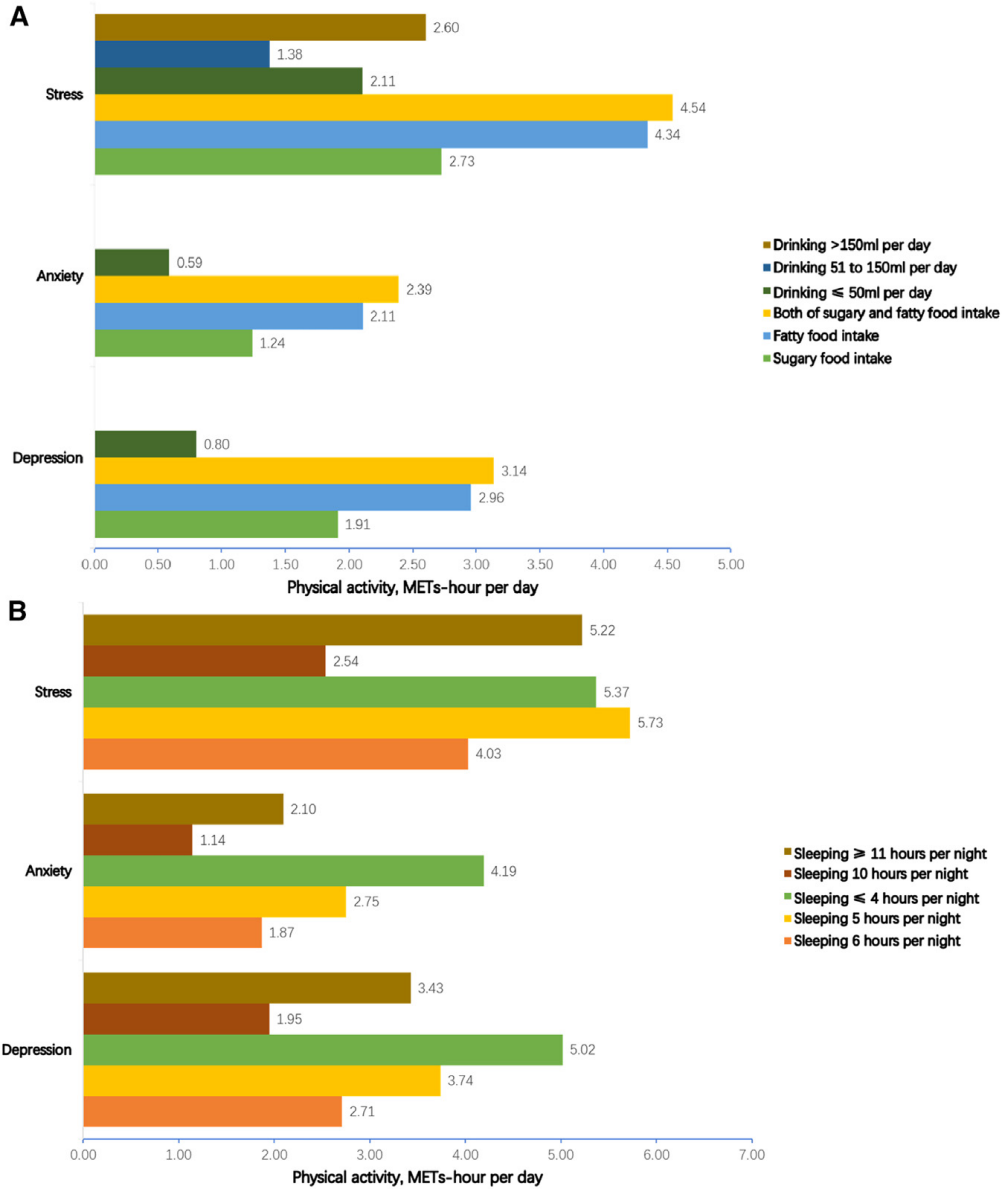
Dose of physical activity to mitigate the adverse effects of risk behaviors from piecewise regression up to thresholds (A) Dose of physical activity to mitigate the adverse effects of unhealthy food intake and drinking; (B) dose of physical activity to mitigate the adverse effects of short and long sleep duration. Data are represented as mitigation dose, D_RB_ = |*β*_RB_/*β*_PA_|. D_RB_ is the dose of physical activity to mitigate the adverse effect of a risk behavior, *β*_RB_ and *β*_PA_ are coefficients of the risk behavior and physical activity, respectively, in the regression model of a specific mental symptom.

Figure 2 shows the dose of physical activity needed to mitigate the effects of risk behaviors on depression, anxiety, and stress. The dose of physical activity was calculated by dividing the *β* value of the risk behavior by that of physical activity when both of the risk behavior and physical activity were significantly associated with the mental health symptom. For example, to mitigate the adverse effects of sugary food intake (>3 times per week) on depression, 1.91 METs-hour per day of physical activity (0.40/0.21) is required. Similarly, 2.96 METs-hour per day (0.62/0.21) and 3.14 METs-hour per day (0.66/0.21) of physical activity could compensate for the effects of fatty food intake and the combined intake of both sugary and fatty food intake (>3 times per week) on depression, respectively. Light drinking (≤50mL per day) would require 0.80 METs-hour per day (0.17/0.21) of physical activity to mitigate its adverse effects on depression (Figure 2A). For the effects of short sleep durations on depression, 2.71 METs-hour per day (0.57/0.21), 3.74 METs-hour per day (0.79/0.21), and 1.05 METs-hour per day (0.62/0.21) of physical activity could compensate for adverse effects of sleeping 6 h, 5 h, and less than 4 h per night, respectively. To mitigate the effects of long sleep durations, 1.95 METs-hour per day (0.41/0.21) and 3.43 METs-hour per day (0.72/0.21) of physical activity are needed for 10 h and 11 h per night, respectively (Figure 2B).

### Results of sensitivity analysis were basically consistent with base case analysis

The results of the subgroup analysis were similar to the main results in the whole sample (Figures S1–S5). The thresholds for physical activity were slightly different for each subgroup, ranging from 1.05 to 2.77 METs-hour per day for depression and anxiety, and 2.15 to 5.45 METs-hour per day for stress.

## Discussion

This study is the first to show dose-response associations between physical activity and several mental health symptoms based on a representative national sample of adults adjusting for risk behaviors. Half of the sample experienced depression, anxiety, or stress symptoms, indicating a concerning increase in mental health problems.^26^ Participants in our study averagely engaged in a moderate level of physical activity, consistent with the WHO recommendations.^7^ Physical activity had a positive effect on mental health outcomes and the effects increased with the dose. Our study also showed that physical activity mitigated the adverse effects of risky behaviors on mental health, providing practical guidance for people with unhealthy habits. These findings might be generalizable to China or other countries facing similar mental health challenges. Additionally, the use of restricted cubic splines and piecewise regression provides a detailed understanding of associations with specific thresholds and doses for mitigation effects, which could inform future interventions.

We observed that physical activity had nonlinear but beneficial associations with common mental health symptoms such as depression, anxiety, and stress, supporting the statement that some physical activity is better than none.^7^ It is well known that physical activity is effective in changing physiological and biochemical indicators, such as body composition, cholesterol and glucose levels, and blood pressure, and leading to better cardiac functions and autonomic balance of the autonomous nervous system, which is essential for improving physical and psychological well-being.^27^ Moreover, physical activity stimulates the brain to release neurotransmitters such as endorphins, dopamine, and hormones such as serotonin, which help reduce symptoms of depression and anxiety.^28^ Additionally, participating in exercises helps people establish positive interpersonal relationships and reduces feelings of loneliness and social anxiety, and enhances self-esteem and self-efficacy through achieving exercise goals and improving physical fitness.^29^

Although there are specific biological and social-psychological mechanisms by which physical activity might positively affect mental health, our study showed that the positive associations between physical activity and mental health symptoms appeared to plateau at low to moderate levels of physical activity. These patterns were consistent with previous studies on psychosomatic complaints of European and North American adolescents,^10^ general mental health in Canada,^9^ and depression in a meta-analysis,^30^ suggesting that although excessive physical activity was not linked to harm, it might not be necessary for mental health. On the other hand, two studies in the US population found a U-shaped association between physical activity and general mental health and suggested that excessive exercise would worsen mental health.^11^^,^^12^ Although there are mixed results of a dose-response relationship between physical activity and mental health among various populations, it can be inferred that excessive physical activity is not necessary for mental health promotion.

In addition, our results confirmed that smokers, drinkers, and people with unhealthy food intake and long sleep duration were more physically active, which was consistent with previous studies indicating co-occurrence of physical activity and some risk behaviors.^23^^,^^24^ The behavioral mechanism why some individuals attempt to compensate for the health hazards posed by unhealthy behaviors might be partially attributed to compensatory belief and behavioral habits and dependencies. When faced with guilt or health concerns stemming from unhealthy behaviors,^31^ people may use physical activity as a psychological compensation to alleviate this pressure and achieve a psychological balance or comfort. Long-formed behavioral habits and dependencies, such as smoking, drinking, eating habits, and sleep duration, are often difficult to change, while physical activity may be seen as a more acceptable and implementable alternative behavior. However, our findings showed that the dose-dependent associations between physical activity and nicotine dependence or alcohol withdrawal anxiety were marginally significant or insignificant. Quit smoking or drinking might be more important and effective than focusing on physical activity to mitigate the addictive symptoms of drinking and smoking.

Meanwhile, we found that physical activity could partially mitigate the adverse effects of risky behaviors on common mental symptoms. For instance, 1 MET-hour per day, equivalent to 20 min of low-intensity physical activity (e.g., walking and mopping),^32^ could approximately counteract the impact of 50mL alcohol drinking on depression and anxiety, or sugary food intake on anxiety; 40 to 60 min of walking could mitigate the negative effects of fatty food intake on depression and anxiety, or 150mL alcohol on stress. People eating both of sugary and fatty food or with extremely short or long sleep duration would need more physical activity to offset the adverse effects, such as 1 h of moderate-intensity physical activity (e.g., jogging and mowing),^33^ or 40 min of high-intensity physical activity (e.g., playing competitive volleyball or farming).^33^ However, it is noteworthy that people with short sleep duration reported less physical activity than people with recommended sleep duration, which posed a challenge to the feasibility of offsetting the risk of short sleep by increasing physical activity.^34^ Moreover, insufficient sleep can increase levels of cardiac troponin T, which raises the risk of cardiovascular events during taking exercises.^35^ These findings suggest that people who have some unhealthy habits could benefit from increasing their physical activity.

In summary, our findings suggest that healthcare professionals should prescribe physical activity based on different mental health symptoms. Engaging in low to moderate levels of physical activity can positively affect common mental health symptoms such as depression, anxiety, and stress. While higher levels of physical activity do no harm to mental health, they may not have additional benefits. For individuals dependent on unhealthy habits, such as sugary and/or fatty food intake, alcohol drinking, and sleeping longer than 9 h, prescribing physical activity ranging from low to high levels can offset the negative effects of those behaviors on common mental symptoms to some degree. Although moderate to high levels of physical activity could compensate for the adverse effects of short sleep duration, it might be difficult for healthcare professionals to change the less active lifestyles of people who lack sleep. Regarding patients suffering from addictive behaviors such as smoking and heavy alcohol drinking, healthcare professionals should address the belief that physical activity can compensate for the negative effects of substance use. Instead, they should focus on reducing their risk behaviors while simultaneously promoting physical activity.^36^

In conclusion, this study identified significant dose-dependent associations between physical activity and common mental health symptoms, as well as the necessary doses to mitigate the effects of risk behaviors. These findings provide valuable insights for designing future interventions to promote mental health in adults, regardless of their current health habits. Future research should focus on investigating the causal relationships between health behaviors and various mental health outcomes through longitudinal or interventional studies. Additionally, developing effective interventions for behavior change will be crucial in enhancing mental health and well-being.

### Limitations of the study

This study has several limitations. First, the cross-sectional nature of this study limits the ability to establish causality between physical activity and mental health, although evidence from longitudinal studies that physical activity offers benefits to symptoms of depression and anxiety could partially mitigate this concern.^7^^,^^37^^,^^38^^,^^39^ However, it is noteworthy that risk behaviors such as physical inactivity, smoking, drinking, and short or long sleep duration might be both contributors and symptoms of mental health issues. The bidirectional nature of the relationship between risk behaviors and mental health underscores the need for future efforts to motivate behavior change.^6^^,^^40^ Therefore, research focusing on exploring influencing factors for mental health-related behaviors based on the theoretical framework of behavior sciences could be a valuable field for future studies. Second, physical activity was self-reported in this study, and therefore recall bias cannot be ruled out. Devise-based measures might be accurate and objective; however, the feasibility of large epidemiological surveys is limited, and the compliance of participants may influence the completion and accuracy of data. Finally, we did not use standardized screening instruments such as the Alcohol Use Disorders Identification Test (AUDIT) to assess the severity of alcohol use, we only have data on the daily amount of alcohol use and a single question on possible alcohol withdrawal, which might affect our findings.

## Resource availability

### Lead contact

Further information and requests for resources should be directed to and will be fulfilled by the lead contact, Feng Jiang (fengjiang@sjtu.edu.cn).

### Materials availability

This study did not generate new unique reagents.

### Data and code availability

• Data: Data used in this study have been deposited at the general-purpose repository DRYAD and are publicly available as of the date of publication at https://doi.org/10.5061/dryad.brv15dvkr.^41^
• Code: All original code has been deposited at DRYAD and is publicly available at https://doi.org/10.5061/dryad.brv15dvkr as of the date of publication.^41^
• Any additional information required to reanalyze the data reported in this article is available from the lead contact upon request, Feng Jiang (fengjiang@sjtu.edu.cn).

## Acknowledgments

This research has been conducted using the data of PBICR in 2023. We thank the participants of the PBICR. Funding: F.J.: The 10.13039/501100012456National Social Science Fund of China (No.23BGL292), and F.J.: National Key Research and Development Program of China (No. 2024YFE0199000).

## Author contributions

Y.W., H.L., and F.J. contributed to the conceptualization of the study. Y.W. and H.Z. did data curation and formal analysis. H.Z., Y-L.T., Y.W., H.L., and F.J. interpreted the data. F.J. and H.L. obtained funding. H.Z. wrote the first draft. Y-L.T. critically revised the article. All authors approved the final version. The corresponding authors attest that all listed authors meet authorship criteria and that no others meeting the criteria have been omitted.

## Declaration of interests

The authors declare no competing interests.

## STAR★Methods

### Key resources table

**Table:** 

REAGENT or RESOURCE	SOURCE	IDENTIFIER
**Deposited data**		

Physical activity and mental health data extracted from the Psychology and Behavior Investigation of Chinese Residents (PBICR)	Published in DRYAD archive	https://doi.org/10.5061/dryad.brv15dvkr.

**Software and algorithms**		

R studio version 4.2.2	R software	https://www.rstudio.com/tags/rstudio/
Detailed code	Published in DRYAD archive	https://doi.org/10.5061/dryad.brv15dvkr.

### Experimental model and study participant details

Data were extracted from the Psychology and Behavior Investigation of Chinese Residents (PBICR), a cross-sectional questionnaire survey among Chinese adults (≥18 years old) conducted from June to August 2023. The study protocol has been published previously.^42^

Using multi-stage and quota sampling methods, PBICR 2023 recruited participants across all 34 administrative regions in China, which include 23 provinces, five autonomous regions, four municipalities, and two special administrative regions. According to the population proportions reported in seventh national census data,^43^ a minimum of 200/500/1000/1500/2000/2500/3000 individuals were sampled from each province/autonomous region/municipality/special administrative region, and the total estimated sample size was 40,000 individuals. Additionally, 2–12 cities were selected from each administrative region, and 10–60 communities were selected from each city according to the ratio of urban to rural communities. Sampling at the individual level was in accordance with the sex and age quota of the country.

Finally, 45,830 participants from 148 cities with 780 communities/villages of the 34 administrative regions were sampled, and 30,054 completed responses were included in the final PBICR 2023 dataset as the national representative sample. All participants volunteered to participate in the face-to-face survey, and signed a consent form.

### Method details

#### Independent variables: Health behaviors

(1) Physical activity: To assess the frequency and duration of physical activity in three types (walking, moderate, and vigorous), the adapted International Physical Activity Questionnaire (IPAQ) short form was used.^44^ According to the IPAQ data processing rules,^45^ we checked maximum and minimum values of duration of activity and found that all 30,054 responses were eligible for analysis. We assigned an average metabolic equivalent (MET) value to each type of physical activity in IPAQ (short form): walking = 3.3 METs, moderate = 4.0 METs, and vigorous = 8.0 METs.^33^ Physical activity scores were calculated by multiplying minutes of physical activity per week by METs of each type. Total physical activity score was the sum of METs for each type. Then the daily average physical activity (METs-hour per day) was calculated and used for analysis.
(2) Unhealthy food intake: Participants were asked to indicate unhealthy food they consumed more than 3 times per week. This variable was categorized as no, sugary food (such as ice-cream, desserts and sugary beverages), fatty food (such as fried food, possessed meat and hot pot), and both of sugary and fatty food;
(3) Drinking: Drinking variable was categorized as no, light (≤50mL per day), moderate (51–150mL per day), and heavy (>150mL per day);
(4) Smoking: Smoking status was categorized as no, light (≤10 cigarettes per day), moderate (11–20 cigarettes per day), and heavy (>20 cigarettes per day).
(5) Sleep duration: Participants were asked how long they usually slept in the past four weeks (not equal to the time in bed)”. They had to select a whole number between 1 and 16 h and round up any half hour. Since sleep duration has a U-shaped association with mental health, and 7–8 h per night is recommended for adults,^46^^,^^47^ we treated sleep duration in a piecewise manner with a breakpoint at 7–8 h. Consequently, short sleep duration variable was categorized as no (7–8 h per night or longer), light (6 h per night), moderate (5 h per night), and extreme (≤4 h per night); long sleep duration variable was categorized as no (7–8 h per night or shorter), light (9 h per night), moderate (10 h per night), and extreme (≥11 h per night).

#### Dependent variables: Mental symptoms

Five symptoms were selected, corresponding to common mental disorders prevalent in China.^48^ The outcome variables were coded as dichotomous ones in our analysis.

(1) Depression: The Patient Health Questionnaire (PHQ-9) was used to assess depression.^49^ The responses were divided into four levels according to the frequency of symptoms in the past two weeks: never = 0 points, several days = 1 point, more than half of the time = 2 points, and almost every day = 3 points. The total score of the scale was 0–27 points. Scoring 5 and above was the cutoff point for having depressive symptoms. The scale has a range of 0–27 points, and a score of 5 or above indicates depressive symptoms.^49^ The Cronbach’s alpha value of these nine items was 0.93, indicating good reliability.^50^
(2) Anxiety: The Generalized Anxiety Disorder (GAD-7) was used to assess anxiety.^51^ The scale also had a high reliability, with a Cronbach’s alpha of 0.94.^50^ The responses were divided into four levels the same as PHQ-9. The total score of GAD-7 ranged from 0 to 21 points. Scoring 5 or more indicates anxiety symptoms.^52^
(3) Stress: The Perceived Stress Scale (PSS-4) was used to assess stress.^53^ The PSS-4 has a Cronbach’s alpha of 0.92).^49^ The responses were divided into five levels according to the frequency of symptoms in the past four weeks from never = 0 points to very often = 4 points. The total score ranged from 0 to 16 points, with higher scores indicating more perceived stress.^54^ Since there is no standard cutoff for stress symptoms, we used the median score as the cutoff for stress symptoms.
(4) Nicotine dependence: The Fagerström Test for Nicotine Dependence (FTND) with six items was used.^55^ It has a Cronbach’s alpha of 0.77 in this survey.^49^ The FTND consists of six items that assess the level of nicotine dependence based on the time to first cigarette after waking up, difficulty refraining from smoking in restricted places, preferred cigarette, daily cigarette consumption, smoking frequency after waking, and smoking while ill. The total score ranges from 0 to 10 points and can be categorized into five levels of nicotine dependence: very low (0–2 points), low (3–4 points), moderate (5 points), high (6–7 points), and very high (8–10 points).^55^ The total score ranges from 0 to 10 points,^56^ and a score of 6 or higher indicates a high level of nicotine dependence.^57^
(5) Alcohol withdrawal anxiety: It was assessed with a binary question: “Do you experience anxiety for at least two days, with most of the time each day, when you stop or reduce drinking?” We used the responses as a categorical variable in our analysis.

#### Covariates

We controlled several potential covariates in our analysis, such as age, sex, education level (primary and middle school, high school, technical school, and university), employment status (employed, unemployed, retired, student, and self-employed), monthly family income per capita (very low <2000 CNY, low 2001–3000 CNY, middle 3001–4000 CNY, high 4001–6000 CNY, and very high >6000 CNY), marital status (married/single), place of residence (rural/urban), presence of any chronic medical disorders (yes/no), and body mass index (BMI, kg/m^2^).

### Quantification and statistical analysis

We applied a two-tailed method for all statistical analyses, and we considered a significance level of 0.05.

We used Statistical Package of the Social Sciences (SPSS version 26; IBM Inc., Armonk, NY, United States) to perform descriptive analysis on the whole study sample, and the subgroups of smokers and alcohol users, respectively. Continuous variables were described as means and standard deviations (SDs), categorical variables were described as numbers (n) and percentages (%). We used the Student’s t test or Chi-square test to compare the characteristics of the sample by five mental health outcomes. We also used the Kruskal-Wallis test to examine physical activity METs by certain risk behaviors.

We applied restricted cubic spline (RCS) regression by package “plotRCS” in R Programming Language (version 4.2.2) to test the nonlinear associations between physical activity scores and five mental health outcomes. We fitted models of depression, anxiety, and stress in the whole sample, a model of nicotine dependence in smokers, and a model of alcohol withdrawal anxiety in alcohol drinkers. If we found evidence of nonlinearity, we estimated a two-line piecewise model mainly by package “segmented” in R Programming Language with a single turning point to quantify the dose-response association.

We fitted simple logistic models for the outcomes that had linear association with physical activity using SPSS. We adjusted all models for risk behaviors (unhealthy food intake, smoking, drinking, short and long sleep duration) and covariates (age, sex, education level, employment status, monthly family income per capita, marital status, place of residence, presence of any chronic medical disorders, and body mass index). We estimated odds ratios d with 95% confidence intervals (CIs) and standardized regression coefficients (β). We calculated the dose of physical activity that could mitigate the adverse effects of risk behaviors by dividing the absolute β values of risk behaviors by that of physical activity when both were significantly associated with mental health outcomes. The equation is as follows:

(Equation 1) $$D_{RB=}|\beta_{RB}÷\beta_{PA}|\;$$

D_RB_ is the dose of physical activity to mitigate the adverse effect of a risk behavior, β_RB_ and β_PA_ are coefficients of the risk behavior and physical activity, respectively, in the regression model of a specific mental symptom.

We conducted sensitivity analysis by performing subgroup analysis by unhealthy food intake, smoking, drinking, and short/long sleep duration to test the robustness of findings.

### Additional resources

The study protocol was registered at the Chinese Clinical Trial Registry compliant with whom on 16 June 2023 (ChiCTR2300072573, protocol link to https://www.chictr.org.cn/showproj.html?proj=197152), and approved by the ethics committees of Shandong Provincial Hospital (SWYX: NO.2023-198).
