# Supplementary material for: Dose-dependent association between physical activity and mental health, and mitigation effects on risk behaviors
Source: iScience. 2025 Jan 23;28(2):111866. doi: 10.1016/j.isci.2025.111866 (PMC11847119; doi:10.1016/j.isci.2025.111866)
Supplement: Document S1. Figures S1–S5 and Tables S1–S4 [file mmc1.pdf]

iScience, Volume *28*

Supplemental information

Dose-dependent association between physical activity and mental health, and mitigation effects on risk behaviors

Huixuan Zhou, Feng Jiang, Huanzhong Liu, Yibo Wu, and Yi-lang Tang

Supplemental Information: Tables S1-S4, and Figures S1-S5.

Table S1. Characteristics of the whole study sample by depression, anxiety and stress (n=30054), Related to RESULTS

	**Total sample (%)**	**Depression**			**Anxiety**			**Stress**		
		**Yes**	**No**	***p value***	**Yes**	**No**	***p value***	**Yes**	**No**	***p value***
**Total sample (%)**	30054 (100)	15435 (51.4)	14619 (48.6)		12575 (41.8)	17479 (58.2)		15851 (52.7)	14203 (47.3)	
**Age (year)** [a]	43.0 ± 16.6	41.2 ± 16.6	44.9 ± 16.3	<0.001	41.1 ± 16.3	44.4 ± 16.6		41.5 ± 16.5	44.7 ± 16.4	
**Sex**				0.09			0.35			0.03
Male	15011 (49.9)	7636 (50.9)	7375 (49.1)		6241 (41.6)	8770 (58.4)		7825 (52.1)	7186 (47.9)	
Female	15043 (50.1)	7799 (51.8)	7244 (48.2)		6334 (42.1)	8709 (57.9)		8026 (53.4)	7017 (46.6)	
**Education level**				<0.001			<0.001			<0.001
Primary or middle school	9243 (30.8)	4020 (43.5)	5223 (56.5)		3297 (35.7)	5946 (64.3)		4477 (48.4)	4766 (51.6)	
High school	6187 (20.6)	3222 (52.1)	2965 (47.9)		2579 (41.7)	36.8 (58.3)		3281 (53.0)	2906 (47.0)	
Professional qualifications	4010 (13.3)	2108 (52.6)	1902 (47.4)		1716 (42.8)	2294 (57.2)		2058 (51.3)	1952 (48.7)	
University	10614 (35.3)	6085 (57.3)	4529 (42.7)		4983 (46.9)	5631 (53.1)		6035 (56.9)	4579 (43.1)	
**Employment status**				<0.001			<0.001			<0.001
Employed	13302 (44.3)	6664 (50.1)	6638 (49.9)		5446 (40.9)	7856 (59.1)		6824 (51.3)	6478 (48.7)	
Students	4681 (15.6)	2903 (62.0)	1778 (38.0)		2308 (49.3)	2373 (50.7)		2902 (62.0)	1779 (38.0)	
Retired	4228 (14.1)	1976 (46.7)	2252 (53.3)		1586 (37.5)	2642 (62.5)		1973 (46.7)	2255 (53.3)	
Self-employed	5043 (16.8)	2497 (49.5)	2546 (50.5)		2093 (41.5)	2950 (58.5)		2686 (53.3)	2357 (46.7)	
Unemployed	2800 (9.3)	1395 (49.8)	1405 (50.2)		1142 (40.8)	1658 (59.2)		1466 (52.4)	1334 (47.6)	
**Monthly family income per capita** [b]				<0.001			<0.001			<0.001
Very low (<2000 CNY)	8781 (29.2)	4752 (54.1)	4029 (45.9)		3859 (43.9)	4922 (56.1)		4910 (55.9)	3871 (44.1)	
Low (2001-3000 CNY)	9460 (31.5)	4651 (49.2)	4809 (50.8)		3770 (39.9)	5690 (60.1)		4611 (48.7)	4849 (51.3)	
Middle (3001-4000 CNY)	3968 (13.2)	2017 (50.8)	1951 (49.2)		1664 (41.9)	2304 (58.1)		2092 (52.7)	1876 (47.3)	
High (4001-6000 CNY)	3491 (11.6)	1677 (48.0)	1814 (52.0)		1362 (39.0)	2129 (61.0)		1783 (51.1)	1708 (48.9)	

Very high (>6000 CNY)	4354 (14.5)	2338 (53.7)	2016 (46.3)		1920 (44.1)	2434 (55.9)		2217 (50.9)	2137 (49.1)	
**Marital status**				<0.001			<0.001			<0.001
Married	19427 (64.6)	8973 (46.2)	10454 (53.8)		7303 (37.6)	12124 (62.4)		9429 (48.5)	9998 (51.5)	
Others	10627 (35.4)	6462 (60.8)	4165 (39.2)		5272 (49.6)	5355 (50.4)		6422 (60.4)	4205 (39.6)	
**Place of residence**				<0.001			<0.001			0.001
Rural	9319 (31.0)	4566 (49.0)	4753 (51.0)		3715 (39.9)	5604 (60.1)		4781 (51.3)	4538 (48.7)	
Urban	20735 (69.0)	10869 (52.4)	9866 (47.6)		8860 (42.7)	11875 (57.3)		11070 (53.4)	9665 (46.6)	
**Presence of any chronic medical disorders**				<0.001			<0.001			<0.001
Yes	9594 (31.9)	5293 (55.2)	4301 (44.8)		5210 (54.3)	4384 (45.7)		5402 (56.3)	4192 (43.7)	
No	20460 (68.1)	10142 (49.6)	10318 (50.4)		8191 (40.0)	12269 (60.0)		10449 (51.1)	10011 (48.9)	
**BMI (kg/m2)** [a]	22.4 ± 3.4	22.3 ± 3.6	22.5 ± 3.2	<0.001	22.3 ± 3.6	22.5 ± 3.3	<0.001	22.3 ± 3.6	22.5 ± 3.3	<0.001
**Physical activity (METs-hour per day)** [a]	4.9 ± 5.8	4.7 ± 5.7	5.0 ± 5.8	<0.001	4.7 ± 5.7	5.0 ± 5.8	<0.001	4.7 ± 5.7	5.0 ± 5.	<0.001
**Unhealthy food intake (>3 times per week)**										
No	17287 (57.5)	7617 (44.1)	9670 (55.9)	<0.001	6071 (35.1)	11216 (64.9)	<0.001	8037 (46.5)	9250 (53.5)	<0.001
Sugary food	5235 (17.4)	3098 (59.2)	2137 (40.8)		2511 (48.0)	2724 (52.0)		3086 (58.9)	2149 (41.1)	
Fatty food	3418 (11.4)	2116 (61.9)	1302 (38.1)		1761 (51.5)	1657 (48.5)		2110 (61.7)	1308 (38.3)	
Both of sugary and fatty food	4114 (13.7)	2604 (63.3)	1510 (36.7)		2232 (54.3)	1882 (45.7)		2618 (63.6)	1496 (36.4)	
**Smoking**				<0.001			<0.001			0.08
No	25116 (83.6)	12733 (50.7)	12383 (49.3)		10325 (41.1)	14791 (58.9)		13197 (52.5)	11919 (47.5)	
Light (≤ 10 cigarettes per day)	2926 (9.7)	1544 (52.8)	1382 (47.2)		1283 (43.8)	1643 (56.2)		1558 (53.2)	1368 (46.8)	
Moderate (11 to 20 cigarettes per day)	1450 (4.8)	806 (55.6)	644 (44.4)		682 (47.0)	768 (53.0)		771 (53.2)	679 (46.8)	

Heavy (> 20 cigarettes per day)	562 (1.9)	352 (62.6)	210 (37.4)		285 (50.7)	277 (49.3)		325 (57.8)	237 (42.2)	
**Drinking**				<0.001			<0.001			<0.001
No	22597 (75.2)	11338 (50.2)	11259 (49.8)		9214 (40.8)	13383 (59.2)		11634 (51.5)	10963 (48.5)	
Light (≤ 50ml per day)	3439 (11.4)	1984 (57.7)	1455 (42.3)		1606 (46.7)	1833 (53.3)		1989 (57.8)	1450 (42.2)	
Moderate (51 to 150ml per day)	1703 (5.7)	877 (51.5)	826 (48.5)		711 (41.7)	992 (58.3)		907 (53.3)	796 (46.7)	
Heavy (>150ml per day)	2315 (7.7)	1236 (53.4)	1079 (46.6)		1044 (45.1)	1271 (54.9)		1321 (57.1)	994 (42.9)	
**Short sleep duration**				<0.001			<0.001			<0.001
No (7-8 hours per night and longer)	22354 (74.4)	10627 (47.5)	11727 (52.5)		8560 (38.3)	13794 (61.7)		11236 (50.3)	11118 (49.7)	
Light (6 hours per night)	5228 (17.4)	3047 (58.3)	2181 (41.7)		2492 (47.7)	2736 (52.3)		3001 (57.4)	2227 (42.6)	
Moderate (5 hours per night)	1288 (4.3)	840 (65.2)	448 (34.8)		703 (54.6)	585 (45.4)		782 (60.7)	506 (39.3)	
Extreme (≤ 4 hours per night)	1184 (3.9)	921 (77.8)	263 (22.2)		820 (69.3)	364 (30.7)		832 (70.3)	352 (29.7)	
**Long sleep duration**				<0.001			<0.001			<0.001
No (7-8 hours per night and shorter)	25861 (86.0)	13293 (51.4)	12569 (48.6)		10842 (41.9)	15019 (58.1)		13647 (52.8)	12214 (47.2)	
Light (9 hours per night)	2745 (9.1)	1258 (45.8)	1487 (54.2)		1005 (36.6)	1740 (63.4)		1345 (49.0)	1400 (51.0)	
Moderate (10 hours per night)	1037 (3.5)	596 (57.5)	441 (42.5)		483 (46.6)	554 (53.4)		585 (56.4)	452 (43.6)	
Extreme (≥ 11 hours per night)	411 (1.4)	288 (70.1)	123 (29.9)		245 (59.6)	166 (40.4)		274 (66.7)	137 (33.3)	

[a] Data are presented as mean ± SD.

[b] According to the China Statistical Yearbook, the average per capita monthly income of Chinese residents in 2022 was 3073 CNY (428 USD).

Table S2. Characteristics of the smoking sample by nicotine dependence, and characteristics of the drinking sample by withdrawal anxiety from drinking, Related to RESULTS

	Smoker	Nicotine dependence			Drinker	Withdrawal anxiety		
		High	Low	*p value*		Yes	No	*p value*
**Total sample (%)**	4938 (100)	1642 (33.3)	3296 (66.7)		7457 (100)	1259 (16.9)	6198 (83.1)	
**Age (years)** [a]	46.0 ± 15.5	47.4 ± 15.1	45.3 ± 15.7	<0.001	41.5 ± 15.6	48.8 ± 15.5	40.0 ± 15.2	<0.001
**Sex**				0.05				<0.001
Male	4411 (89.3)	1447 (32.8)	2964 (67.2)		5653 (75.8)	1082 (19.1)	4571 (80.9)	
Female	527 (10.7)	195 (37.0)	332 (63.0)		1804 (24.2)	177 (9.8)	1627 (90.2)	
**Education level**				0.22				<0.001
Primary or middle school	1755 (35.5)	614 (35.0)	1141 (65.0)		2025 (27.2)	558 (27.6)	1467 (72.4)	
High school	1155 (23.4)	376 (32.6)	779 (67.4)		1556 (20.9)	250 (16.1)	1306 (83.9)	
Professional qualifications	803 (16.3)	267 (33.3)	536 (66.7)		1068 (14.3)	135 (12.6)	933 (87.4)	
University	1225 (24.8)	385 (31.4)	840 (68.6)		2808 (37.7)	316 (11.3)	2492 (88.7)	
**Employment status**				0.001				<0.001
Employed	2425 (49.1)	763 (31.5)	1662 (68.5)		3684 (49.4)	522 (14.2)	3162 (85.8)	
Students	338 (6.8)	92 (27.2)	246 (72.8)		1214 (16.3)	76 (6.3)	1138 (93.7)	
Retired	618 (12.5)	220 (35.6)	398 (64.4)		702 (9.4)	209 (29.8)	493 (70.2)	
Self-employed	1105 (22.4)	394 (35.7)	711 (64.3)		1364 (18.3)	315 (23.1)	1049 (76.9)	
Unemployed	452 (9.2)	173 (38.3)	279 (61.7)		493 (6.6)	137 (27.8)	356 (72.2)	
**Monthly family income per capita** [b]				0.001				<0.001
Very low (<2000 CNY)	1556 (31.5)	576 (37.0)	980 (63.0)		2010 (27.0)	447 (22.2)	1563 (77.8)	
Low (2001-3000 CNY)	1498 (30.3)	477 (31.8)	1021 (68.2)		2268 (30.4)	396 (17.5)	1872 (82.5)	
Middle (3001-4000 CNY)	612 (12.4)	201 (32.8)	411 (67.2)		965 (12.9)	144 (14.9)	821 (85.1)	
High (4001-6000 CNY)	542 (11.0)	178 (32.8)	364 (67.2)		923 (12.4)	124 (13.4)	799 (86.6)	
Very high (>6000 CNY)	730 (14.8)	210 (28.8)	520 (71.2)		1291 (17.3)	148 (11.5)	1143 (88.5)	

**Marital status**				0.11				<0.001
Married	3480 (70.5)	1133 (32.6)	2347 (67.4)		4696 (63.0)	924 (19.7)	3772 (80.3)	
Others	1458 (29.5)	509 (34.9)	949 (65.1)		2761 (37.0)	335 (12.1)	2426 (87.9)	
**Place of residence**				<0.001				<0.001
Rural	1767 (35.8)	652 (36.9)	1115 (63.1)		2119 (28.4)	519 (24.5)	1600 (75.5)	
Urban	3171 (64.2)	990 (31.2)	2181 (68.8)		5338 (71.6)	740 (13.9)	4598 (86.1)	
**Presence of any chronic medical disorders**				<0.001				<0.001
Yes	2054 (41.6)	789 (38.4)	1265 (61.6)		2460 (33.0)	648 (26.3)	1812 (73.7)	
No	2884 (58.4)	853 (29.6)	2031 (70.4)		4997 (67.0)	611 (12.2)	4386 (87.8)	
**BMI (kg/m^2)** [a]	23.1 ± 3.5	23.1 ± 3.7	23.1 ± 3.4	0.90	23.0 ± 3.5	23.1 ± 3.4	22.9 ± 3.5	0.10
**Physical activity (METs-hour per day)** [a]	5.5 ± 6.6	5.5 ±7.0	5.5 ± 6.4	0.71	5.7 ± 6.6	5.5 ± 6.7	5.7 ± 6.6	0.32
**Unhealthy food intake (>3 times per week)**								
No	2539 (51.4)	714 (28.1)	1825 (71.9)	<0.001	3760 (50.4)	665 (17.7)	3095 (82.3)	0.19
Sugary food	893 (18.1)	337 (37.7)	556 (62.3)		1308 (17.5)	198 (15.1)	1110 (84.9)	
Fatty food	749 (15.2)	281 (37.5)	468 (62.5)		1141 (15.3)	188 (16.5)	935 (83.5)	
Both of sugary and fatty food	757 (15.3)	310 (41.0)	447 (59.0)		1248 (16.7)	208 (16.7)	1040 (83.3)	
**Smoking**				<0.001				<0.001
No	N/A	N/A	N/A		4601 (61.7)	505 (11.0)	4096 (89.0)	
Light (≤ 10 cigarettes per day)	2926 (59.3)	583 (19.9)	2343 (80.1)		1631 (21.9)	387 (23.7)	1244 (76.3)	
Moderate (11 to 20 cigarettes per day)	1450 (29.4)	650 (44.8)	800 (55.2)		897 (12.0)	252 (28.1)	645 (71.9)	
Heavy (> 20 cigarettes per day)	562 (11.4)	409 (72.8)	153 (27.2)		328 (4.4)	115 (35.1)	213 (64.9)	
**Drinking**				<0.001				<0.001
No	2082 (42.2)	660 (31.7)	1422 (68.3)		N/A	N/A	N/A	
Light (≤ 50ml per day)	936 (19.0)	253 (27.0)	683 (73.0)		3439 (46.1)	285 (8.3)	3154 (91.7)	
Moderate (51 to 150ml per day)	766 (15.5)	280 (36.6)	486 (63.4)		1703 (22.8)	369 (21.7)	1334 (78.3)	
Heavy (>150ml per day)	1154 (23.4)	449 (38.9)	705 (61.1)		2315 (31.0)	605 (26.1)	1710 (73.1)	

**Short sleep duration**				<0.001				<0.001
No (7-8 hours per night and longer)	3212 (65.0)	949 (29.5)	2263 (70.5)		5370 (72.0)	835 (15.5)	4535 (84.5)	
Light (6 hours per night)	932 (18.9)	321 (34.4)	611 (65.6)		1385 (18.6)	236 (17.0)	1149 (83.0)	
Moderate (5 hours per night)	251 (5.1)	94 (37.5)	157 (62.5)		335 (4.5)	59 (17.6)	276 (82.4)	
Extreme (≤ 4 hours per night)	543 (11.0)	278 (51.2)	265 (48.8)		367 (4.9)	129 (35.1)	238 (64.9)	
**Long sleep duration**				<0.001				0.02
No (7-8 hours per night and shorter)	4365 (88.4)	1462 (33.5)	2903 (66.5)		6535 (87.6)	1077 (16.5)	5458 (83.5)	
Light (9 hours per night)	351 (7.1)	88 (25.1)	263 (74.9)		582 (7.8)	105 (18.0)	477 (82.0)	
Moderate (10 hours per night)	136 (2.8)	46 (33.8)	90 (66.2)		234 (3.1)	50 (21.4)	184 (78.6)	
Extreme (≥ 11 hours per night)	86 (1.7)	46 (53.5)	40 (46.5)		106 (1.4)	27 (25.5)	79 (74.5)	

[a] Data are presented as mean ± SD.

[b] According to the China Statistical Yearbook, the average per capita monthly income of Chinese residents in 2022 was 3073 CNY (428 USD).

Table S3. Association between physical activity and nicotine dependence in smokers below and above the threshold, Related to RESULTS

	PA≤4.26 (n=2836)					PA>4.26 (n=2102)				
	**β**	***p value***	**OR**	**Lower 95%CI**	**Upper 95%CI**	**β**	***p value***	**OR**	**Lower 95%CI**	**Upper 95%CI**
**PA, METs-hour per day**	-0.07	0.047	0.94	0.88	1.00	0.02	0.005	1.02	1.01	1.03
**Unhealthy food intake, ref. no**										
Sugary food	0.46	<0.001	1.58	1.23	2.02	0.37	0.016	1.45	1.07	1.97
Fatty food	0.36	0.006	1.44	1.11	1.87	0.51	0.001	1.67	1.23	2.26
Both of sugary and fatty food	0.61	<0.001	1.83	1.41	2.39	0.60	<0.001	1.82	1.32	2.51
**Smoking, ref. light (≤ 10 cigarettes per day)**										
Moderate (11 to 20 cigarettes per day)	1.15	<0.001	3.17	2.62	3.83	1.27	<0.001	3.54	2.82	4.45
Heavy (> 20 cigarettes per day)	2.13	<0.001	8.42	6.41	11.06	2.55	<0.001	12.85	9.03	18.29
**Drinking, ref. no**										
Light (≤ 50ml per day)	0.14	0.240	1.16	0.91	1.47	-0.16	0.320	0.85	0.62	1.17
Moderate (51 to 150ml per day)	0.20	0.119	1.23	0.95	1.58	0.33	0.038	1.38	1.02	1.88
Heavy (>150ml per day)	0.22	0.057	1.25	0.99	1.56	0.22	0.099	1.25	0.96	1.63
**Short sleep duration, ref. no**										
Light (6 hours per night)	0.25	0.031	1.29	1.02	1.62	0.14	0.338	1.15	0.87	1.51
Moderate (5 hours per night)	0.18	0.375	1.20	0.81	1.78	0.09	0.712	1.09	0.69	1.73
Extreme (≤ 4 hours per night)	0.31	0.040	1.36	1.01	1.82	1.04	<0.001	2.83	1.85	4.33
**Long sleep duration, ref. no**										
Light (9 hours per night)	-0.07	0.681	0.93	0.65	1.32	-0.20	0.388	0.82	0.51	1.30
Moderate (10 hours per night)	0.16	0.562	1.17	0.69	1.98	0.60	0.057	1.82	0.98	3.38
Extreme (≥ 11 hours per night)	0.50	0.118	1.64	0.88	3.06	1.69	<0.001	5.41	2.41	12.14

The estimates were adjusted for covariates. PA, physical activity; MET, metabolic equivalent; ref., reference group; OR, odds ratio; CI, confidence interval.

Table S4. Association between physical activity and alcohol withdrawal anxiety in drinkers (n=7457), Related to RESULTS

	β	*p value*	OR	Lower 95%CI	Upper 95%CI
**PA, METs-hour per day**	-0.01	0.114	0.99	0.98	1.00
**Unhealthy food intake, ref. no**					
Sugary food	0.13	0.202	1.14	0.93	1.38
Fatty food	0.10	0.331	1.10	0.91	1.34
Both of sugary and fatty food	0.31	0.002	1.37	1.12	1.67
**Smoking, ref. no**					
Light (≤ 10 cigarettes per day)	0.55	<0.001	1.73	1.47	2.03
Moderate (11 to 20 cigarettes per day)	0.67	<0.001	1.95	1.62	2.36
Heavy (> 20 cigarettes per day)	0.87	<0.001	2.38	1.82	3.12
**Drinking, ref. light (≤ 50ml per day)**					
Moderate (51 to 150ml per day)	0.72	<0.001	2.05	1.71	2.45
Heavy (>150ml per day)	1.04	<0.001	2.83	2.40	3.34
**Short sleep duration, ref. no**					
Light (6 hours per night)	0.14	0.124	1.15	0.96	1.37
Moderate (5 hours per night)	0.13	0.430	1.13	0.83	1.55
Extreme (≤ 4 hours per night)	0.76	<0.001	2.14	1.64	2.79
**Long sleep duration, ref. no**					
Light (9 hours per night)	0.18	0.154	1.20	0.94	1.53
Moderate (10 hours per night)	0.58	0.001	1.79	1.25	2.56
Extreme (≥ 11 hours per night)	0.63	0.011	1.88	1.16	3.05

The estimates were adjusted for covariates. PA, physical activity; MET, metabolic equivalent; ref., reference group; OR, odds ratio; CI, confidence interval.

Figure S1. Dose-response relationships between physical activity and depression in subgroup samples from restricted cubic spline regression, Related to RESULTS

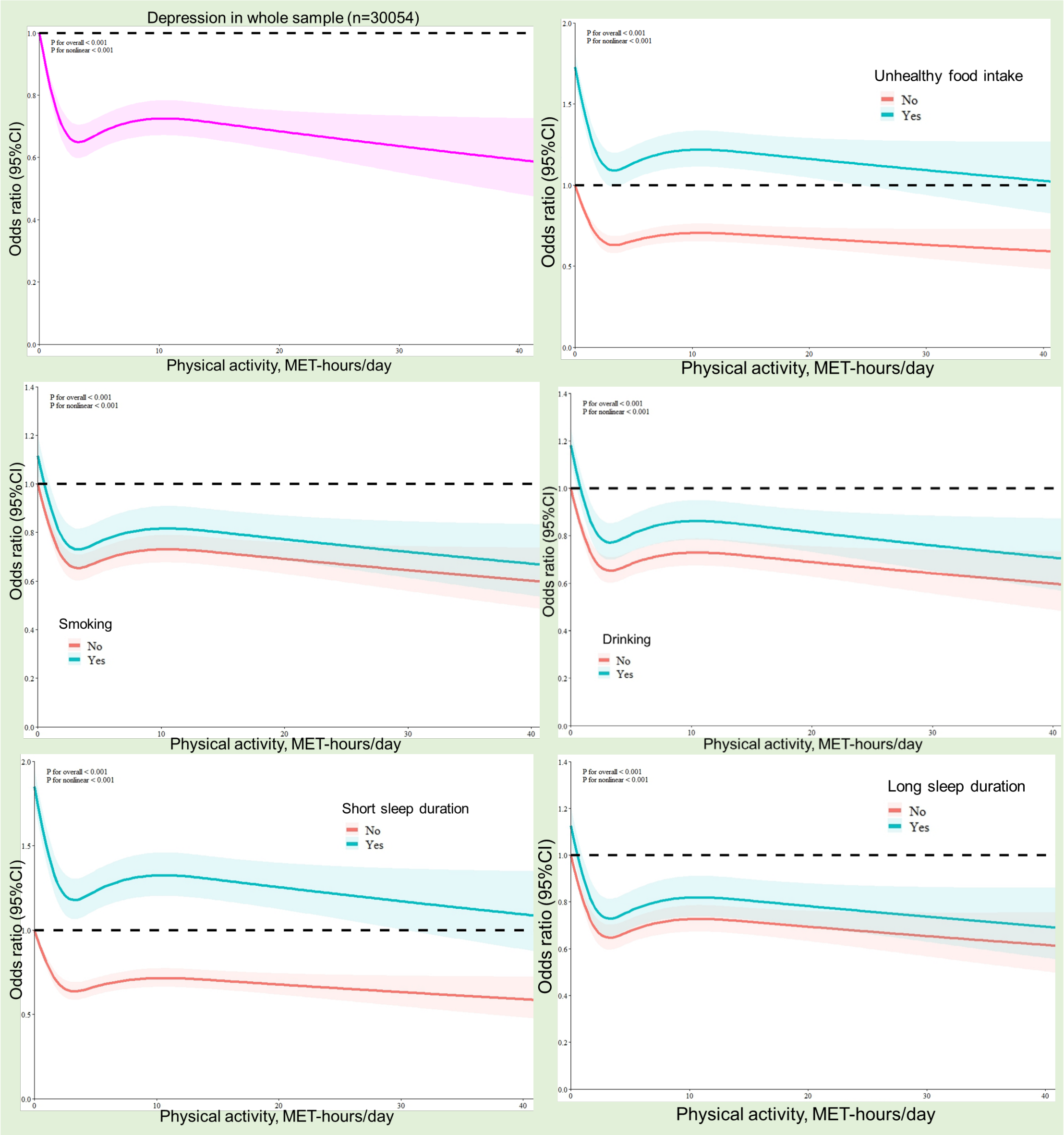

The estimates were adjusted for covariates and risk behaviors.

Figure S2. Dose-response relationships between physical activity and anxiety in subgroup samples from restricted cubic spline regression, Related to RESULTS

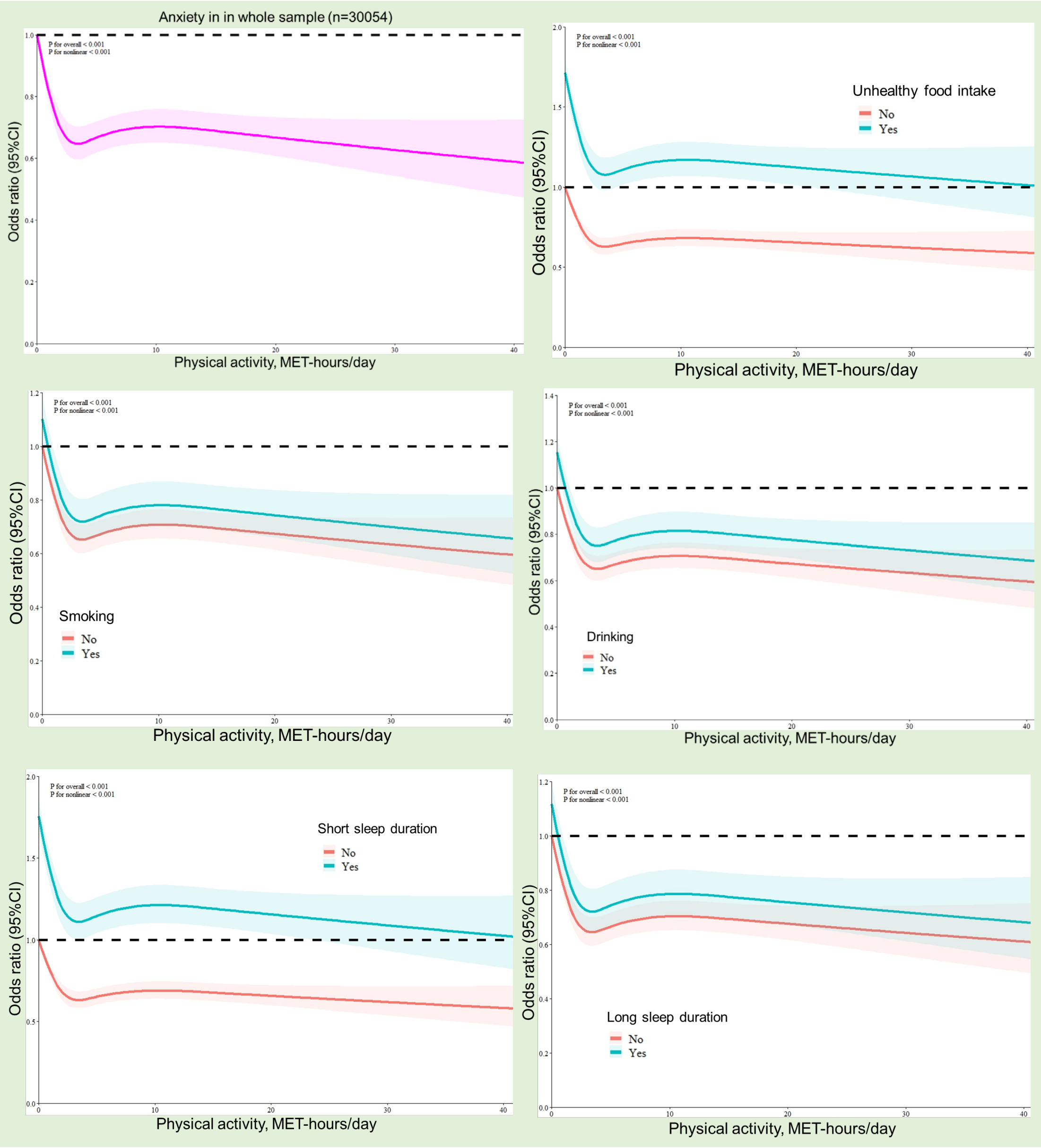

The estimates were adjusted for covariates and risk behaviors.

Figure S3. Dose-response relationships between physical activity and stress in subgroup samples from restricted cubic spline regression, Related to RESULTS

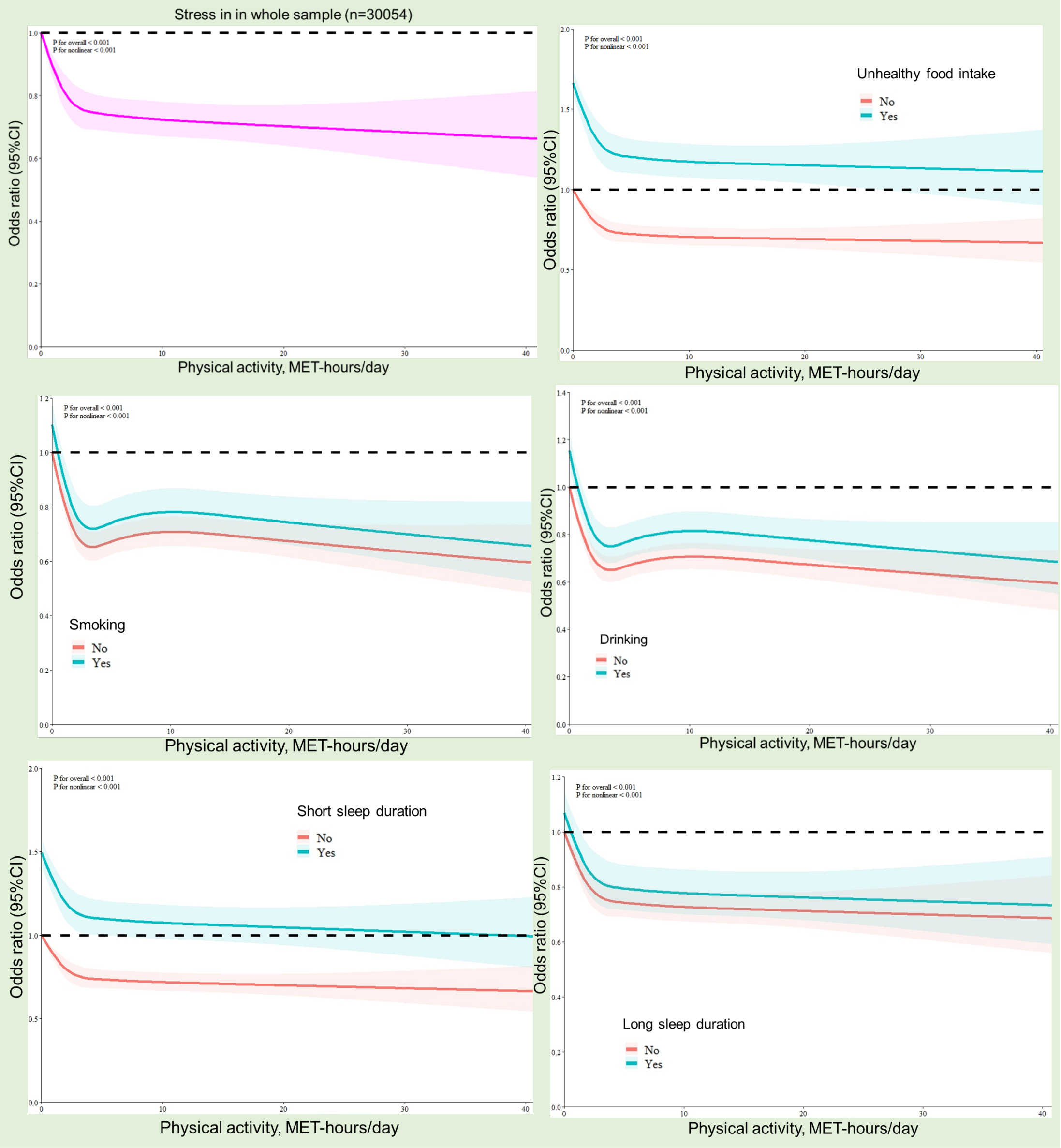

The estimates were adjusted for covariates and risk behaviors.

Figure S4. Dose-response relationships between physical activity and nicotine dependence in subgroup samples from restricted cubic spline regression, Related to RESULTS

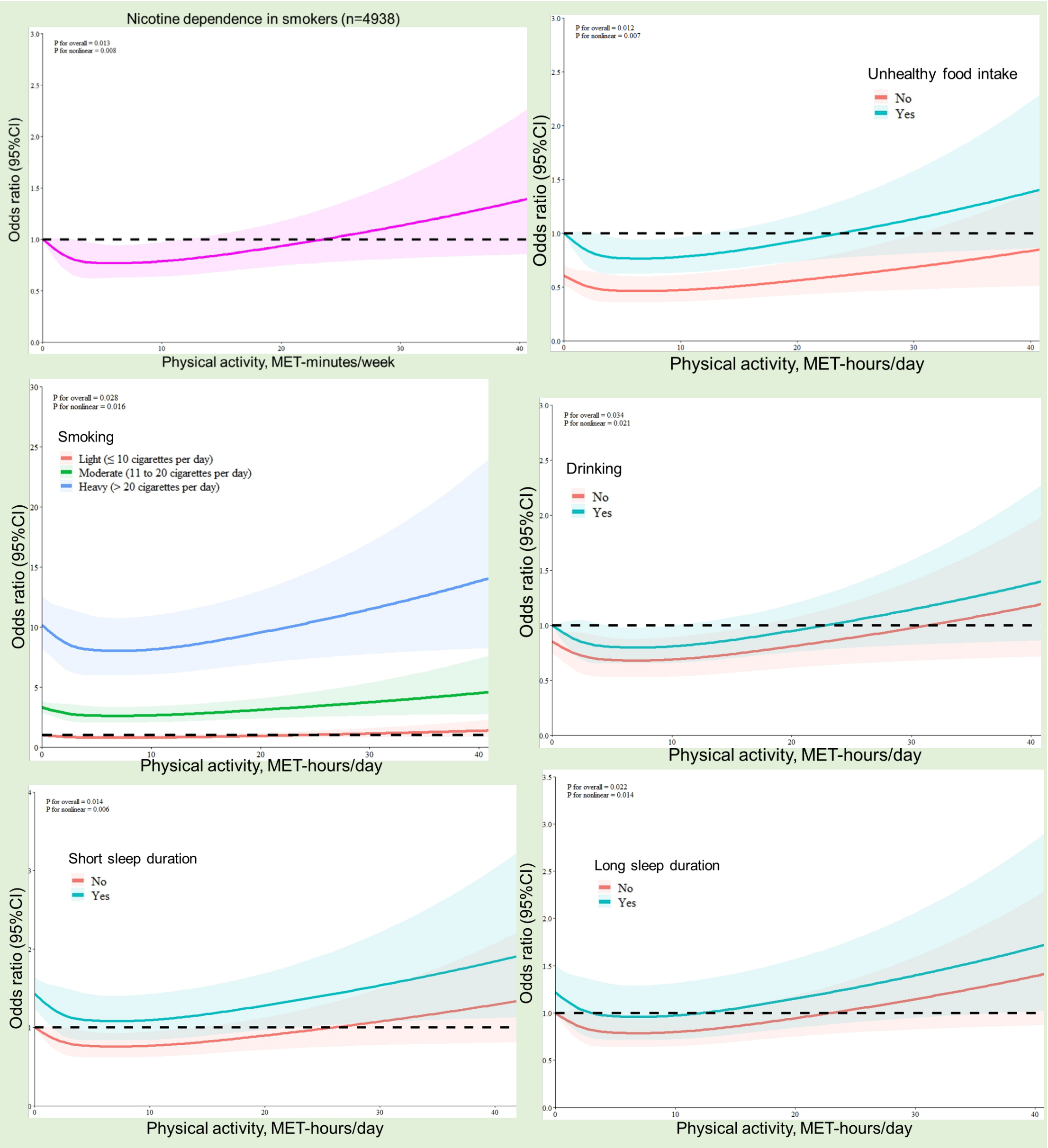

The estimates were adjusted for covariates and risk behaviors.

Figure S5. Dose-response relationships between physical activity and alcohol withdrawal anxiety in subgroup samples from restricted cubic spline regression, Related to RESULTS

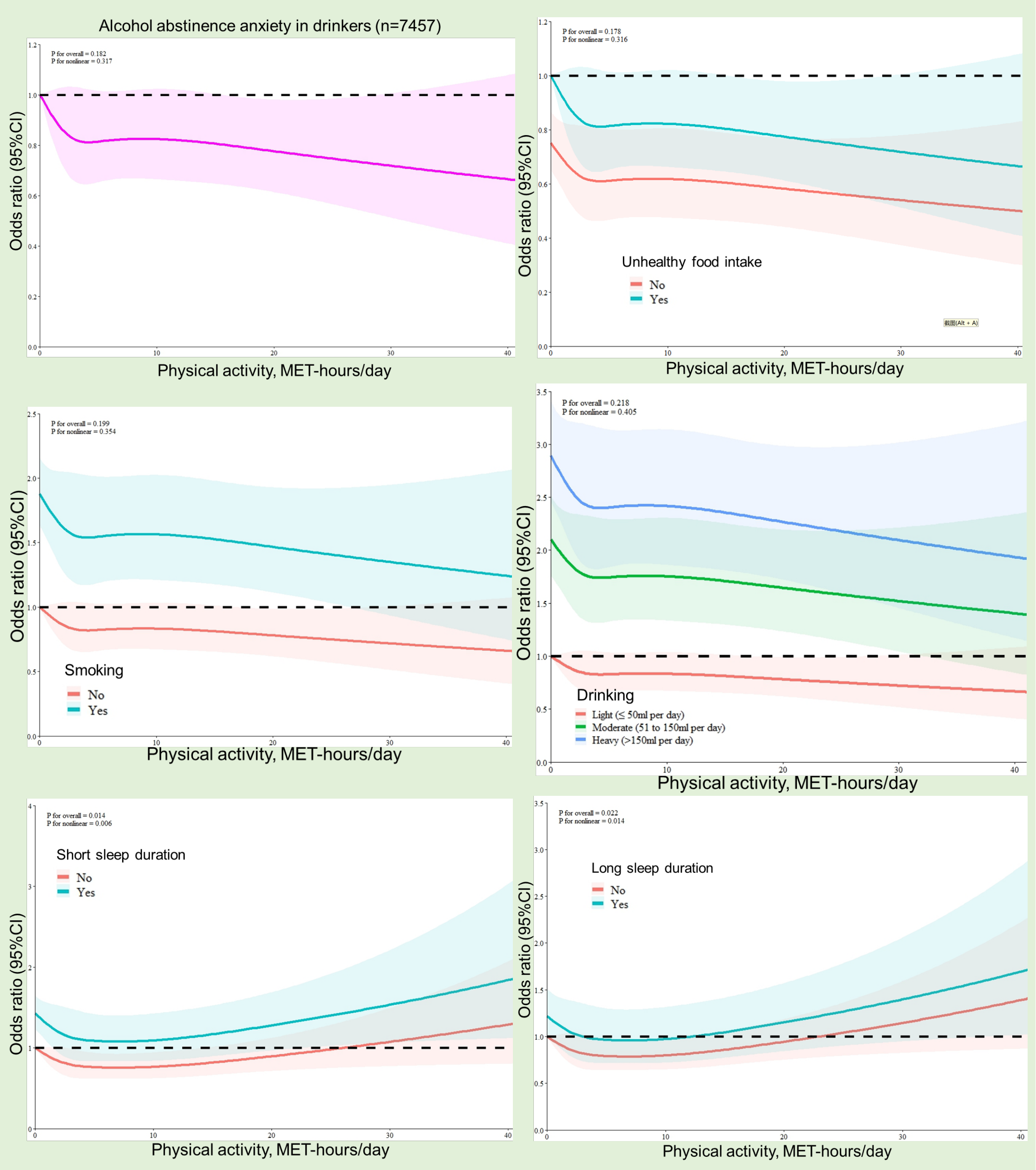

The estimates were adjusted for covariates and risk behaviors.
